# Measuring Utility of VASS Elder Mistreatment Screener Within Community Dwelling U.S. Chinese Older Adults

**DOI:** 10.1093/geroni/igab046.3713

**Published:** 2021-12-17

**Authors:** Natalie Tuseth, XinQi Dong, Stephanie Bergren

**Affiliations:** 1 Rutgers Institute for Health, Health Care Policy and Aging Research, New Brunswick, New Jersey, United States; 2 Rutgers, New Brunswick, New Jersey, United States; 3 Rutgers University, New Brunswick, New Jersey, United States

## Abstract

Elder mistreatment (EM) is often underreported, making potential screening a valuable tool. There is limited literature on the screening utility, especially for minority populations. This abstract aims to study sensitivity and specificity of a commonly used 10-point EM screener compared to a detailed EM questionnaire among Chinese older adults. This study used data from a representative sample of 3,157 community-dwelling U.S. Chinese older adults 60+. Chi-squared test was conducted between VASS 10-questionare screener and EM measured by 56 items on psychological, physical and sexual mistreatment, caregiver neglect and financial exploitation subtypes. Sensitivity and specificity was calculated using the Bayes Theorem. In this sample, average age was 72 and 59% female. 637 (20.30%) reported any EM while 475 (15.14%) older adults screened positive for EM. Of participants reporting any EM, 365 (57.30%) did not screen positive for EM. The screener had a sensitivity of 42.70% and specificity of 91.88% for all EM subtypes. Gaps between reported EM and negative EM screener is smaller in psychological (sensitivity 72.85%, specificity 91.07%) and physical (sensitivity 63.64%, specificity 86.66%) EM subtypes, but much larger in financial exploitation (sensitivity 34.60%, specificity 86.85%) and neglect (sensitivity 14.11%, specificity 84.75%). The VASS screener demonstrates poor sensitivity but acceptable specificity rate for any EM. The screener showed better sensitivity and specificity for physical and psychological mistreatment, but performed worse for more common forms of mistreatment like financial exploitation and neglect. Modifying this screener may improve sensitivity and specificity in identifying EM.

